# Lung Ultrasound in the Critically Ill Neonate

**DOI:** 10.2174/157339612802139389

**Published:** 2012-08

**Authors:** Daniel A Lichtenstein, Philippe Mauriat

**Affiliations:** 1 Service de Réanimation, Hôpital Ambroise-Paré, F-92100 Boulogne (Paris-Ouest), France; 2 Service d'Anesthésie-Réanimation II, Hôpital Haut-Lévèque, 33600 Pessac, France

**Keywords:** BLUE-protocol, critical ultrasound, lung ultrasound, neonate intensive, care, pneumonia, pneumothorax, pulmonary edema.

## Abstract

Critical ultrasound is a new tool for first-line physicians, including neonate intensivists. The consideration of the lung as one major target allows to redefine the priorities. Simple machines work better than up-to-date ones. We use a microconvex probe. Ten standardized signs allow a majority of uses: the bat sign (pleural line), lung sliding and the A-line (normal lung surface), the quad sign and sinusoid sign indicating pleural effusion regardless its echogenicity, the tissue-like sign and fractal sign indicating lung consolidation, the B-line artifact and lung rockets (indicating interstitial syndrome), abolished lung sliding with the stratosphere sign, suggesting pneumothorax, and the lung point, indicating pneumothorax. Other signs are used for more sophisticated applications (distinguishing atelectasis from pneumonia for instance...). All these disorders were assessed in the adult using CT as gold standard with sensitivity and specificity ranging from 90 to 100%, allowing to consider ultrasound as a reasonable bedside gold standard in the critically ill. The same signs are found, with no difference in the critically ill neonate. Fast protocols such as the BLUE-protocol are available, allowing immediate diagnosis of acute respiratory failure using seven standardized profiles. Pulmonary edema e.g. yields anterior lung rockets associated with lung sliding, making the B-profile. The FALLS-protocol, inserted in a Limited Investigation including a simple model of heart and vessels, assesses acute circulatory failure using lung artifacts. Interventional ultrasound (mainly, thoracocenthesis) provides maximal safety. Referrals to CT can be postponed. CEURF proposes personnalized bedside trainings since 1990. Lung ultrasound opens physicians to a visual medicine.

## INTRODUCTION

The use of ultrasound for immediate management of life-threatening conditions is one among the main changes of these last decades. Since its first use in the medical field [1], ultrasound has recently been used in current medical disciplines, including the critically ill patient, the emergency room, etc. 

Neonatal care is a priority target for a physician. The lung is the main vital organ. Assessing lung function in neonates is therefore a major concern. Traditional tools raise issues: CT, too irradiating and requiring transportation, cannot be used as a routine gold standard. Bedside radiography is regularly used for lack of anything better, but to our knowledge no work has assessed its real value. Radiography is of major help when finding unexpected changes [2-4], yet correct assessment of negative findings, as well as of positive findings, is not established. 

Could lung ultrasound be conceivable, given the traditional view that it is of little relevance in assessing air-containing organs [5]? If this were possible, all the well-known advantages of ultrasound would come into play: bedside diagnosis, avoidance of irradiation, and cost-effectiveness. 

We have worked as intensivists in the medical ICU of François Jardin. Since it was equipped with an ultrasound machine (for heart assessment), at a time when most ICUs did not have this option (1985), we have been in a privileged position for discovering and defining the wide field of critical ultrasound. The lung appeared as the priority target. Our most time-consuming task was to publish the concept of lung ultrasound in the critically ill. Its value has now been confirmed by many teams [6-32]. 

The 1982 technology we first used was sufficient for defining, at the bedside, the potential of critical ultrasound [33]. 

## THE SEVEN PRINCIPLES OF LUNG ULTRASOUND

Lung ultrasound in the critically ill is based on seven simple principles. 

The first principle is that of simplicity. The best machine for practicing lung ultrasound is probably the simplest. We have used since 1992 a gray-scale, cost-effective machine (without Doppler), which is still manufactured, with a cathode-ray tube giving optimal image resolution. Our unit is 29 cm wide. If modern laptop machines are larger, they will be less easy to carry to the bedside. Lung ultrasound examination is achieved using natural images, avoiding filters, especially those designed to suppress artifacts. The probe is a critical part of the unit. We use a microconvex probe that can easily be applied everywhere (including heart, belly, optic nerve...) and gives satisfactory image quality from the surface to deep within the tissues (in the adult, from 1 to 17 cm). A compact machine (flat keyboard) and the use of a single probe are important in ensuring easy cleaning, i.e., safe use, which is of prime importance in pediatrics.

The second principle notes that air and fluids are mixed within the thorax. From this mingling arise the main relevant artifacts. The patient’s position should be specified, since air and fluids find their positions according to gravity. 

The third principle recalls that the lung is the most voluminous organ, even in the neonate. We have proposed standardized points of analysis for our fast protocols, using landmarks valid at any age, from adult to neonate, to define two anterior points, and one semiposterior point of strategic relevance [34]. 

**Fig. (1):**
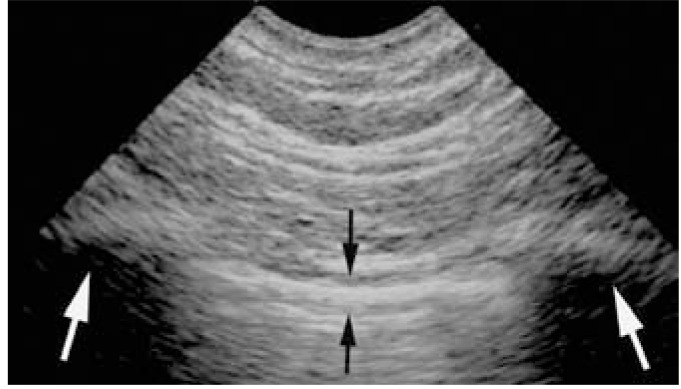
**The bat sign.** The pleural line, the first sign of standardized lung ultrasound. The white arrows indicate the shadow of the upper and lower ribs. The dark arrows indicate the exact level of the pleural line. This pattern is called the bat sign, since one can imagine the wings and body of a bat (a long-time user of ultrasound), provided longitudinal scans are performed. The bat pattern has the same proportions in adults and neonates.

The fourth principle indicates that all signs of lung ultrasound arise from the pleural line. Using a microconvex probe and a longitudinal view, the ribs are recognized and generate posterior shadows. Below the rib line, i.e., 1/2 cm in the adult, but in the same proportion in the neonate, is the pleural line. The whole designates the standardized bat sign (Fig. **1**). 

**Fig. (2):**
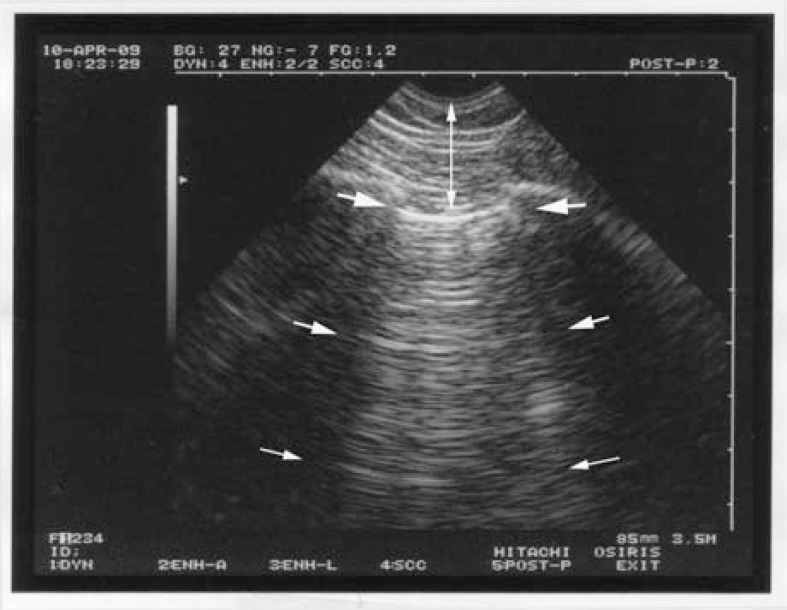
**The A-line.** Arising from the pleural line (upper and larger arrows), two reflections of the pleural line are visible (middle and lower arrows). They are equidistant. The distance between two A-lines is equal to the skin-pleural line distance (vertical arrow). A-lines are the expression of air, i.e., normal alveolar air or free air of a pneumothorax.

The fifth principle specifies that lung ultrasound is mainly based on artifacts—those structures traditionally deemed undesirable. The basic normal artifact, called the A-line, is a horizontal line parallel to the pleural line on the screen (Fig. **2**). A-lines are the demonstration of air. 

**Fig. (3):**
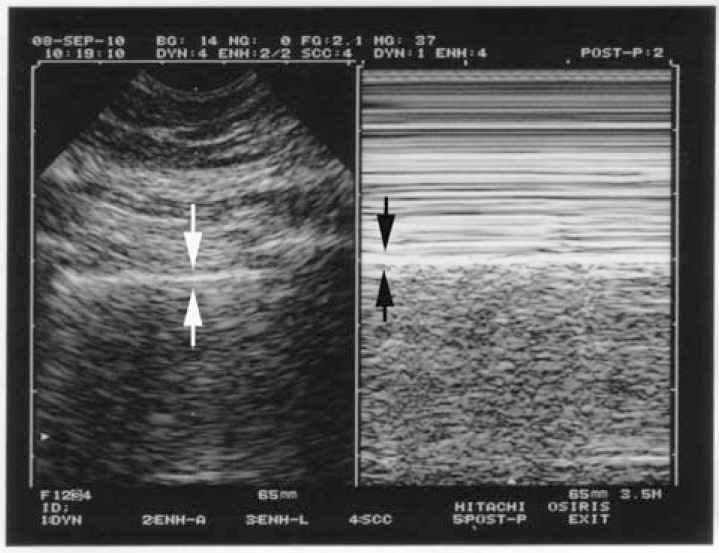
**The seashore sign (lung sliding).** The normal pleural line shows a permanent movement which spreads homogeneously downwards. This results, in M-mode (right image), in a sandy pattern, arising exactly from the pleural line. Above the pleural line is a regular pattern, completely distinct from the sandy pattern seen below. The seashore sign is a simple way to display lung sliding on a frozen view. Note that both images (left, real-time, right, M-mode) are located at the very same level, a mandatory condition for a machine intended for use with lung ultrasound.

According to the sixth principle, lung ultrasound is a dynamic science. Like any vital organ, the lung is in permanent movement. This generates lung sliding, a kind of twinkling visible at the pleural line and spreading homogeneously below, generating in M-mode a standardized pattern, the seashore sign (Fig. **3**).

**Fig. (4):**
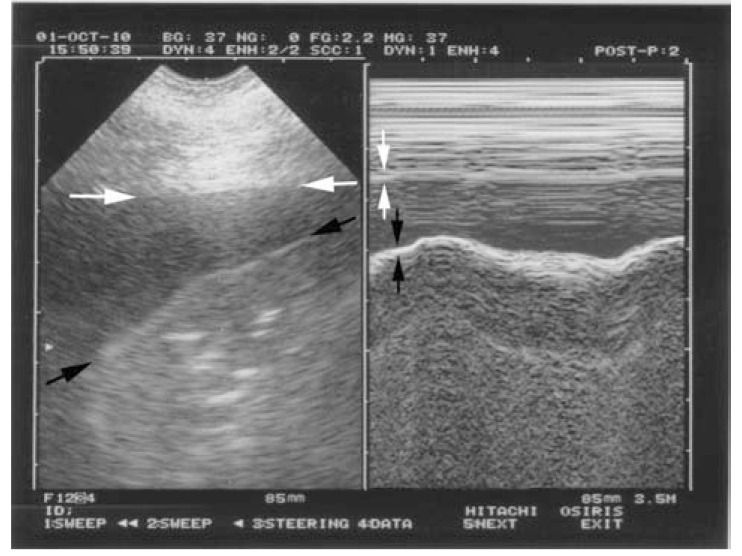
**The quad sign and the sinusoid sign (pleural effusion).** Left: real time, showing the quad sign. The pleural effusion is delineated by four regular borders: the pleural line (white arrows), the shadow of the ribs (not seen here), and mostly the lung line (black arrows), which demonstrates the lung surface (visceral pleura), always regular, and roughly parallel to the pleural line. Right: M-mode, showing the sinusoid sign. The lung line (black arrows) moves toward the pleural line (white arrows) on inspiration. The quad sign and sinusoid sign are universal, as opposed to the anechoic pattern of the effusion, which is applicable only to uncomplicated effusions. They are highly sensitive and quite specific.

**Fig. (5):**
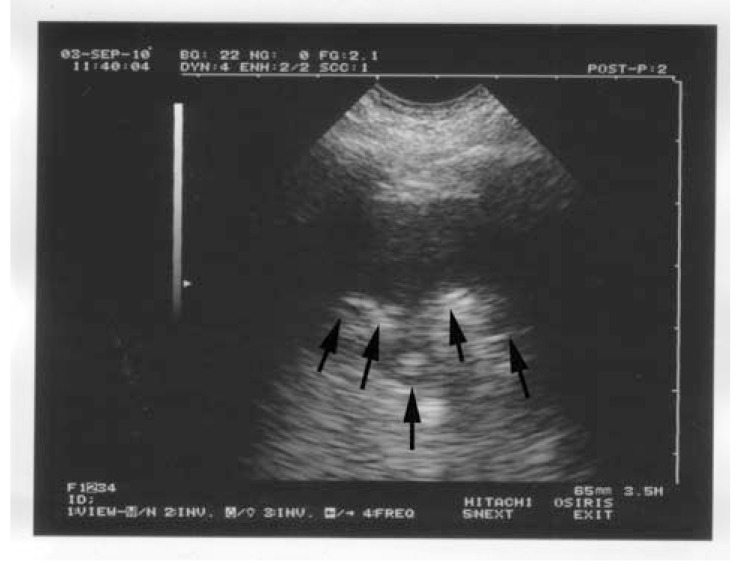
**The shred sign (alveolar syndrome).** Real image arising from the pleural line. In spite of its anechoic tone (mimicking a pleural effusion according to the traditional definitions), the deep limit (arrows) is shredded. The shred sign is quite specific to lung consolidation.

**Fig. (6):**
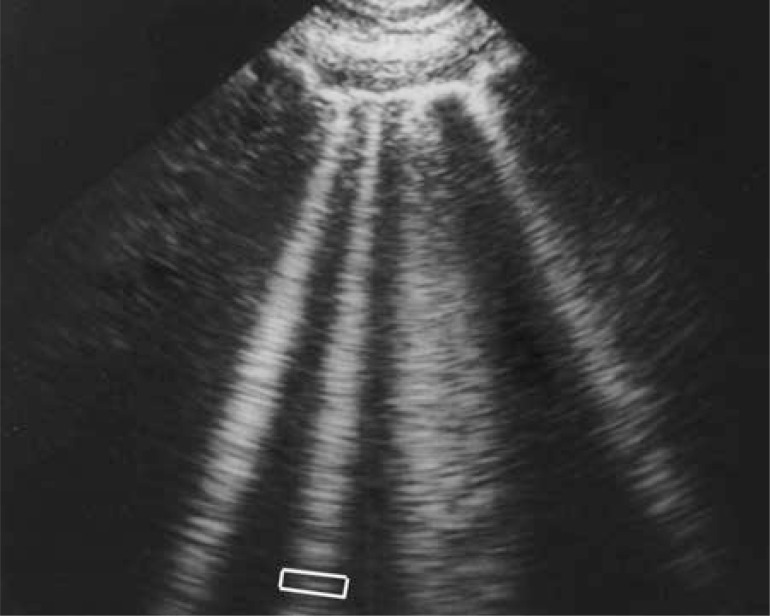
**Lung rockets (interstitial syndrome).** This sign is highly relevant in acute lung ultrasound in the critically ill. It shows here four or five B-lines. The B-line is a comet-tail artifact, arising from the pleural line, hyperechoic like the pleural line, spreading out without fading to the edge of the screen, well-defined, erasing the A-lines, and moving in concert with lung sliding. Three or more B-lines are called lung rockets, and are equivalent to interstitial syndrome. They are used to differentiate the different types of acute respiratory failure, and as help in managing acute circulatory failure. In the frame, one J-line (among many) is isolated, showing that the B-line is a vertical line shaped by numerous small horizontal lines.

**Fig. (7):**
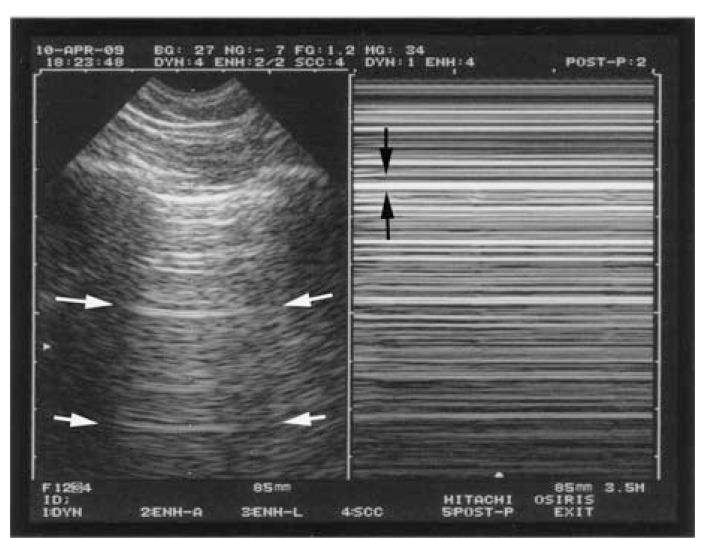
**The stratosphere sign (pneumothorax).** Pneumothorax. On the left, this real-time image is frozen and cannot display abolished lung sliding, yet the use of M-mode (right image) clearly shows the absolute absence of movement at the level of the pleural line (black arrows): the stratosphere sign. Note again on the left image the A-line sign, indicating that only A-lines can be visualized in pneumothorax (white arrows). This again indicates air (see Fig. **2**).

**Fig. (8):**
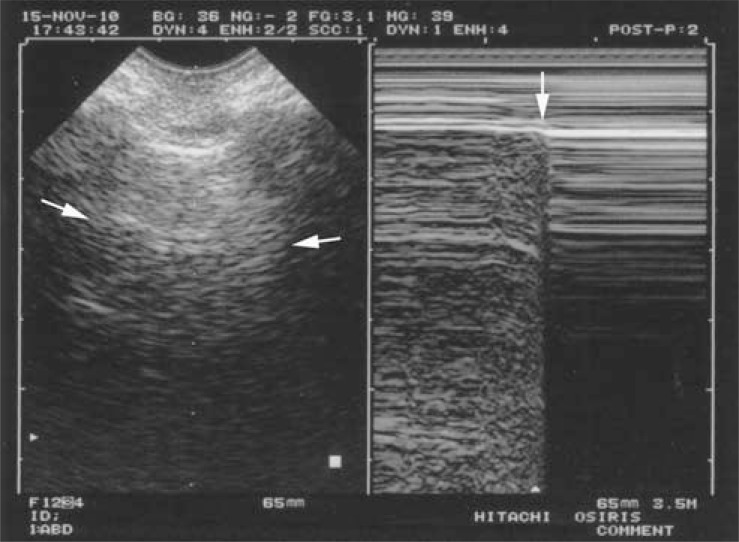
**The lung point (pneumothorax).** Left (real-time), only an ill-defined (but sufficient) A-line is visible. Right, M-mode shows that in a particular area of the chest wall, the lung is (before the vertical arrow) or is no longer (after the arrow) in contact with the pleural line in a cyclic rhythm. This sudden appearance, or disappearance, of lung signs, the lung point, is specific to pneumothorax.

The seventh principle observes that all acute disorders needing immediate care abut the pleura, and are therefore accessible to ultrasound. We will just analyze four basic disorders: pleural effusion, lung consolidation, interstitial syndrome, and pneumothorax. All cases of pneumothorax and pleural effusion reach the pleura, the huge majority (98.5%) of consolidations reach the wall [35], and acute thickening of interlobular septa is a diffuse phenomenon, which always reaches the visceral pleura [36]. The location is standardized: anterior points for pneumothorax and interstitial syndrome of clinical relevance, the semiposterior point for all pleural effusions and 90% of lung consolidations [35]. Pleural effusion, first described long ago [1, 37], can be identified with two standardized signs, making diagnosis possible even for echoic collections (Fig. **4**): the *quad sign, *which points up the fact that the lung is regular in shape and roughly parallel to the chest wall, hence creating a regular line: the lung line. And the *sinusoid sign, *which indicates the movement of the lung line toward the pleural line [38]. Lung consolidation, long described through its classic tissue-like image [39], yields a standardized sign, the *shred sign*, which indicates that the deep limit of this image is irregular (shredded), as opposed to the smooth lung line of pleural effusions (Fig. **5**) [35]. Interstitial syndrome is one of the most useful applications in the acutely ill patient. The air-fluid mingling generates a comet-tail artifact labeled the *B-line*, with seven criteria (read Fig. **6** caption), making it a standardized sign [36, 40]. Several B-lines between two ribs are called lung rockets and define interstitial syndrome. The diagnosis of pneumothorax can also be made using a standardized approach, provided sequential thinking is followed. First, complete *abolition of lung sliding* at the anterior chest wall in supine or semirecumbent patients, a sensitive sign, described long ago in horses [41] - but not specific [42]. Abolished lung sliding generates the standardized *stratosphere sign* [43] (Fig. **7**). Second, only A-lines can be visible facing a pneumothorax: the *A-line sign*. One B-line is sufficient to confidently rule out pneumothorax at this area [44]. Abolished lung sliding and the A-line sign are highly suggestive of pneumothorax, but not sufficient. The third sign, the *lung point,* is specific. When the probe detects abolished lung sliding and the A-line sign at the anterior wall, it then moves gradually toward lateral and if needed posterior areas, until a sudden change of pattern is observed, in sync with the respiratory cycle, with appearance of lung sliding and/or B-lines [45] (Fig. **8**). The lung point location indicates the volume of the pneumothorax. Many other applications will not be dealt with here (such as phrenic assessment). 

## MAJOR POTENTIAL OF LUNG ULTRASOUND

From our reading of the last four principles, it appears that not more than ten signs can be described for basic use in the critically ill. This ten-note scale can be enlarged to a twelve-note scale using the dynamic air bronchogram [46] and the lung pulse [47]. It can also be reduced to a seven-note scale in the BLUE-protocol, with the concept of the PLAPS (PosteroLateral Alveolar and/or Pleural Syndrome), an onomatopoeic term describing either a lung consolidation or a pleural effusion, or both, located posteriorly or laterally, with the advantage that describing effusion instead of consolidation and vice versa does not change the decision tree of the BLUE-protocol. PLAPS are usually sought at the PLAPS-point. 

Once mastered, these signs allow an infinite range of applications: thoracentesis in ventilated patients for diagnostic or therapeutic purposes, airway management, diagnosis and management of pneumothorax, including assessment of volume and progression, diagnosis and follow-up of pneumonia, assessment of ARDS, noninvasive assessment of acute dyspnea in neonates, partial solution of the problem of cost in areas with limited resources, help in various fields (emergency room, pulmonology and cardiology departments, thoracic surgery, family medicine...). We will outline the BLUE-protocol and the FALLS-protocol, two settings where interstitial syndrome plays the main role. 

## THE BLUE-PROTOCOL: IMMEDIATE DIAGNOSIS OF THE CAUSE OF ACUTE RESPIRATORY FAILURE

The BLUE-protocol is an ultrasound approach using lung and venous findings to deal with the daily question of the cause of acute respiratory failure. The two aims are to expedite the relief of the patient and to decrease exposure to radiation. Tests such as CT and arterial puncture become less relevant once the BLUE-protocol has established the cause of acute respiratory failure. This protocol is part of a simple approach that considers the patient’s history, the physical examination and basic laboratory findings (white cells, D-dimers etc). In the adult, where it was created, it can diagnose in less than 3 minutes the six main causes seen in 97% of patients: pneumonia, hemodynamic pulmonary edema, asthma, COPD, pulmonary embolism, and pneumothorax [48]. It confirms older studies on the potential of diagnosing pulmonary edema [49]. 

Examination of the three standardized lung areas is used to draw “BLUE-profiles”, which combine signs with their location, one of the original features of the BLUE-protocol. Each of the seven profiles gives one diagnosis. Broadly speaking, the B-profile (diffuse anterior lung rockets plus lung sliding) indicates hemodynamic pulmonary edema (sensitivity 97%, specificity 95%). The A-profile (predominant anterior A-lines plus lung sliding) suggests pulmonary embolism and mandates testing for venous thrombosis. The A-profile plus venous thrombosis is 81% sensitive and 99% specific to embolism in acute respiratory failure. In the absence of visible thrombosis, the A-profile associated with PLAPS indicates pneumonia (sensitivity 42%, specificity 96%), or, if PLAPS are absent (i.e., normal examination), acute asthma or COPD with 89% sensitivity and 97% specificity. Other typical profiles for pneumonia are the C-profile (anterior consolidation), with 21% sensitivity and 99% specificity, the A/B profile (unilateral anterior lung rockets), with 14% sensitivity and 100% specificity, and the B’-profile (diffuse anterior lung rockets with abolished lung sliding), with 11% sensitivity and 100% specificity. Pneumothorax yields the A’-profile (absence of anterior B-line plus abolished lung sliding). 

All these combinations are easier than they appear for a novice simultaneously discovering the signs and the applications of lung ultrasound. 

Several questions regarding the BLUE-protocol are frequently asked: Why is the heart not featured in the decision tree? Is the BLUE-protocol difficult to learn? Is the average timing of three minutes really possible? What should one think of the missed cases, of the patients excluded because there is no final diagnosis, or double diagnoses, or rare diseases? How about mild cases seen in the emergency room? How about challenging (overweight) patients? Will the BLUE-protocol work everywhere in the world? How does one manage patients with pulmonary embolism but without visible venous thrombosis? Can the BLUE-protocol distinguish between hemodynamic and permeability-induced pulmonary edema?. Answers to these and other questions can be found in [50]. 

In pediatric care, the BLUE-protocol should be slightly adapted, using epidemiological considerations, but the basis remains the same. 

## THE FALLS-PROTOCOL: MANAGEMENT OF ACUTE CIRCULATORY FAILURE

Assessing circulatory status is difficult, especially in the neonate. Traditional tools (Swan-Ganz catheterization, transesophageal echocardiography, PICCO) cannot be used. And even if they could, nobody can affirm that one is better than another, in the absence of a solid gold standard. In association with, or if necessary as a replacement for, the usual approach, the FALLS-protocol (Fluid Administration Limited by Lung Sonography) proposes a simple parameter, which has major advantages, when considering the A-lines and the B-lines. B-lines appear when the pulmonary artery occlusion pressure reaches 18mmHg [51]. At this stage, the pulmonary edema is interstitial, not yet alveolar, and is clinically quiet. The change from A-lines to B-lines during fluid therapy is an on-off parameter, suggesting that the B-line is a *direct marker* of clinical volemia. Interstitial edema is the first, clinically silent step and the FALLS-protocol takes advantage of this pathophysiological event [52]. 

Our “Limited Investigation” considering hemodynamic therapy includes first simple cardiac sonography, to rule out obstructive shock (tamponade, right ventricle enlargement in pulmonary embolism, tension pneumothorax). Then the lungs spirit of the BLUE-protocol, to rule out cardiogenic shock (see B-profile). When there is an A-profile, one can consider that the patient can receive fluid therapy: the FALLS-protocol begins. Patient improvement on fluid therapy is a therapeutic test which defines hypovolemic shock. Bearing in mind that obstructive, cardiogenic and hypovolemic causes have been eliminated, shock that resists fluid therapy, with a lung initiating interstitial syndrome (B-profile appearing on fluid therapy) defines septic shock. This is the time to initiate vasopressor therapy in this patient who has been managed from the beginning according to current standards: early and massive fluid therapy [53]. All these sequences are schematic and must be adapted in the light of clinical assessment. 

## LUNG ULTRASOUND APPLIED TO THE NEONATE 

In a 3-year observational study in a large Parisian neonate ICU, we made two major findings. First, all ten signs described and assessed in the adult using CT were seen again, with no difference [54]. Second, discrepancies appeared between ultrasound and radiography. We invite the reader to consider the contribution of lung ultrasound, through use of its standardized semiotics, and then to form an opinion concerning these discrepancies. 

In the adult, ultrasound has proved more accurate than radiography (Tables **1** and **2**) and nearly as accurate as CT [35, 36, 38, 42-45, 55], and superior on occasion [56]. We must consider that the radiological signs for the main acute disorders are the same in adults and neonates, as no radiological distinction has ever been made [57]. There is no pathophysiological reason for considering that these disorders would give different signs in neonates and adults [58]. In some CT observations in neonates, we observed no difference for pneumothorax, pleural effusion or lung consolidation. In the frequent case where bedside radiography finds no alveolar syndrome, but ultrasound clearly describes a shred sign, we invite the reader to review the radiograph again, to pay more attention to subtle signs, and to admit that reading a bedside radiograph calls for careful attention and involves a great deal of subjectivity. We ask the question: Which disorder other than consolidation can simulate a shred sign? If the answer is *none*, it will be indisputable proof of ultrasound’s superiority. In adults, the use of CT regularly shows the superiority of ultrasound over X-rays in these kinds of questions. 

The potential weakness of bedside radiography is explainable by its principle: three dimensions are reduced to two. Therefore, summations are generated, creating an expert, interpreter-dependent discipline. Alveolar, pleural and interstitial signs can be difficult to distinguish. Anterior interstitial syndrome is not easy to recognize if associated with posterior alveolar lesions (not a problem using ultrasound, which works in three dimensions, and can distinguish anterior from posterior changes). Retrodiaphragmatic alveolar or pleural disorders are missed (not a problem using ultrasound). Our observations suggest that radiography has better specificity than sensitivity. What is not seen can exist anyway. We have on one side a subjective and hazardous technique (at least in the adult). On the other side, a standardized method. It is true, radiography gives an overview (unlike ultrasound) and ultrasound is reputed to be operator-dependent, yet this operator-dependency is easily solved by standardized training. 

## VARIOUS ADVANTAGES OF LUNG ULTRASOUND

Ultrasound in the critically ill was long reduced to echocardiography and abdominal scanning. The inclusion of the lung redefines the field. Using the FALLS-protocol (lung ultrasound for hemodynamic assessment), echocardiography can be simplified. 

The feasibility of lung ultrasound is high, since the pleural line, from which all signs arise, is superficial. The most voluminous organ cannot be missed (unlike the heart). 

The standardization of lung ultrasound makes it easier than most disciplines. Unlike the abdomen, where nearly 20 organs have to be assessed, there is only one organ. Unlike the heart or obstetrical ultrasound, the normal lung pattern is defined using only two signs, wherever the probe is applied. This generates a high interobserver concordance when training benefits from our standardized approach (currently taught by the CEURF) [35]. This question touches on the classic medicolegal issues of critical ultrasound, which hamper its widespread use, but which can be reversed in the near future. We have elegant answers to the issue of time-dependent situations, of increasing radiation [59-62] mainly.

The number of limitations of lung ultrasound is dramatically reduced if a standardized technique is used. Artifacts from subcutaneous emphysema can mimic B-lines, but the bat sign is missing. Parasite comet-tail artifacts arising from the pleural line (mainly Z-lines) do not meet the seven criteria of the B-line. Abdominal fat can mimic lung consolidation, but the use of standardized points of analysis prevents this confusion. Intrathoracic fluid may come from an ectopic stomach, but in this case there is no lung line, no quad sign. Echoic pleural effusions may mimic lung consolidations, but they can easily be distinguished by consideration of the lung line (*vs*. shred line). Lung sliding abolished by inappropriate filters is not a pitfall, provided filters and modes suppressing artifacts are not used. Pleural symphysis with abolished lung sliding and the A-line sign cannot be confused with a pneumothorax, since it never generates any lung point. Ghost artifacts mimic juxtaphrenic disorders through the traditional subcostal approach, but never using the intercostal approach. Hypertrophied thymus cannot be confused with a lung consolidation, since not generating any shred sign. Deep disorders (lymph nodes) are not a concern in acute settings. In other words, the real limitations are not numerous: rare cases of consolidation not abutting the pleura, extensive dressings, and subcutaneous emphysema. 

## CONCLUSIONS

Lung ultrasound in the neonate is a small part of lung ultrasound, which is subsumed by critical ultrasound, itself just one aspect of medical ultrasound. This small part may change habits, especially for those working in intensive care of neonates. As regards ultrasound, the lung of the neonate is a miniature adult lung. To detect the basic signs and then use them for infinite applications, the seven principles of lung ultrasound should be followed, mainly the principle of simplicity. Then ultrasound provides a different way of management, opening up a whole new world of visual medicine. 

## Figures and Tables

**Table 1.** Published Performance of Ultrasound Compared with CT

Ultrasound	Sensitivity	Specificity
Pleural effusion [ref. 38]	94%	97%
Alveolar consolidation [ref. 35]	90%	98%
Interstitial syndrome [ref. 36]	93%	93%
Pneumothorax [ref. 45]	95%	94%
Complete pneumothorax [ref. 44]	100%	96%
Occult pneumothorax [ref. 43]	79%	100%

**Table 2.** Accuracy of Radiography in Critically Ill Adults [55]

	Sensitivity	Specificity
Pleural effusion	39%	85%
Alveolar consolidation	68%	95%
Interstitial syndrome	60%	100%
